# Nested structure of intraspecific competition network in *Carnobacterium maltaromaticum*

**DOI:** 10.1038/s41598-020-63844-5

**Published:** 2020-04-30

**Authors:** Nancy E. Ramia, Cécile Mangavel, Claire Gaiani, Aurélie Muller-Gueudin, Samir Taha, Anne-Marie Revol-Junelles, Frédéric Borges

**Affiliations:** 1 Université de Lorraine, LIBio, F-54000 Nancy, France; 2 Laboratoire de Biotechnologies Appliquées, EDST, Université Libanaise, Tripoli, Lebanon; 3 Université de Lorraine, CNRS, Inria, IECL, F-54000 Nancy, France

**Keywords:** Bacteria, Microbial ecology

## Abstract

While competition targeting food-borne pathogens is being widely documented, few studies have focused on competition among non-pathogenic food bacteria. *Carnobacterium maltaromaticum* is a genetically diverse lactic acid bacterium known for comprising several bacteriocinogenic strains with bioprotective potentialities against the food-borne pathogen *Listeria monocytogenes*. The aim of our study is to examine the network properties of competition among a collection of 73 strains of *C. maltaromaticum* and to characterize their individual interaction potential. The performed high-throughput competition assays, investigating 5 329 pairwise interactions, showed that intraspecific competition was major in *C. maltaromaticum* with approximately 56% of the sender strains antagonizing at least one receiver strain. A high diversity of inhibitory and sensitivity spectra was identified along with a majority of narrow inhibitory as well as sensitivity spectra. Through network analysis approach, we determined the highly nested architecture of *C. maltaromaticum* competition network, thus showing that competition in this species is determined by both the spectrum width of the inhibitory activity of sender strains and the spectrum width of the sensitivity of receiver strains. This study provides knowledge of the competition network in *C. maltaromaticum* that could be used in rational assembly of compatible microbial strains for the design of mixed starter cultures.

## Introduction

Microbial competition is a widespread phenomenon in food ecosystems, especially in fermented foods where microbial biomass is high and where resource limitation occurs, thus leading to the promotion of competition^1^. Two types of competition can be distinguished: exploitation competition and interference competition. Exploitation competition involves the relatively more efficient use of a limiting resource while interference competition implies the production of substances that impair the growth or the survival of competitors^2^. Competition has long been applied for food biopreservation purposes which is mainly based on the use of microorganisms capable of inhibiting food-borne pathogens by interference competition *via* secreting antagonistic substances such as bacteriocins^3–6^. Hence, studies on microbial competition in food have led to an increased knowledge of competition between bioprotective food microorganisms and unwanted food microorganisms^7,8^.

However, the ecology of competition in general, and more specifically competition between nonpathogenic food microorganisms, is not well known. A better understanding of the role of competition on the assembly, the structure, and the evolution of food microbial communities would allow a better control of food ecosystems and more specifically would help to implement new approaches allowing to design functional food microbiomes.

Under certain circumstances, competition does not necessarily lead to the reduction of diversity as it was experimentally shown for *Escherichia coli*^9^. For this species, an equilibrium could be established in a three-actors’ model in which three bacterial strains coexisted, with one strain producing a bacteriocin, the second one being sensitive to the bacteriocin and the third being resistant to it. This scenario can be mapped to the children’s game RPS (rock–paper–scissors), a game with no single winning strategy^9^. Similar results were obtained with *in silico* models. Several actors were able to cohabit if the density of antagonistic interactions was high along with a high diversity of the produced substances and a high diversity of the interactive roles played by the different interacting actors^10^. Nonetheless, these models are both considered as bottom-up approaches^11^ meaning that the models only consider small subpopulations of microorganisms that are not representative of more complex competition networks involving a larger number of microorganisms.

For a better understanding of microbial competition, approaches based on network analysis were recently used to analyze the statistical properties of microbial competition networks^12^. These networks were studied by performing pairwise competition assays between strains of a collection. For each test, one strain, called the “sender”, was tested for its antagonistic potential against another strain, called the “receiver”, whose sensitivity was addressed^13^. Competition assays were performed in such a way that each strain was tested (i) as sender against all the other strains of the collection tested as receivers, and (ii) as receiver against all the other strains of the collection tested as senders. Therefore, for a collection of *n* strains, this required to perform *n*^2^ competition assays. Analysis of the competition network in *Streptomyces* revealed that the structure of the competition network was determined by the antagonistic potential of the sender strains, rather than the resistance level of receiver strains^13^. A network analysis approach was also used to better understand the effect of temperature variations on the outcome of competition interactions in arctic bacteria^14^. In *Bacillus*, network analysis revealed that antagonism is a determining factor in the spatial exclusion of conspecific strains^15^.

While bacterial competition networks are being widely investigated in models of environmental bacteria, the structure of competition networks among food bacteria remains unknown. The aim of this study was to assess the intraspecific antagonistic capacity of the lactic acid bacterium *C. maltaromaticum*. *C. maltaromaticum* is a generalist LAB (Lactic Acid Bacterium) that can be found in various environmental and food products such as meat, sea and dairy products, as well as in the gastroinstestinal tract^16,17^. This species is characterized by a high genetic diversity^18,19^ and known for the anti-*Listeria monocytogenes* properties of several strains thanks to the production of bacteriocins. In a previous study investigating interactions among representative species of bacteria isolated from vacuum-packaged (VP) beef, it was shown that *C. maltaromaticum* was capable of inhibiting a wider range of isolates compared to other tested LAB^20^. *C. maltaromaticum* is therefore an interesting model to study intraspecific competition. The potential competition network was studied within a collection of 73 strains, using a high-throughput approach, followed by a network analysis aiming to identify the resulting network architecture.

## Results

**Figure 1:**
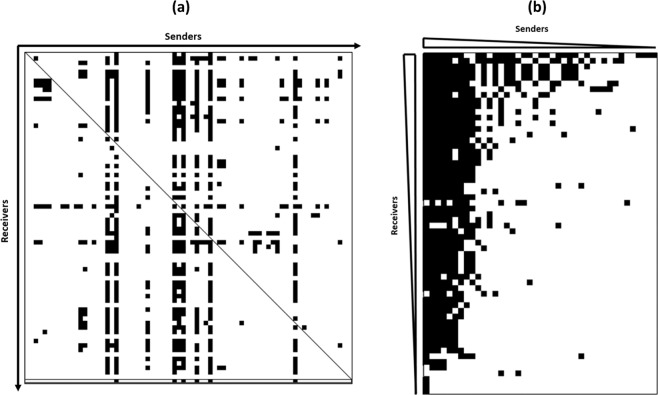
(**a**) Heatmap representing the pairwise interaction matrix between the 73 tested *C. maltaromaticum* strains*. X*-axis depicts the tested strains in the position of senders and *Y*-axis depicts the tested strains in the position of receivers. In addition, the last line which is framed in black lines, shows the inhibitory activity against *L. monocytogenes* EGDe. Black cells represent antagonistic interactions while white cells represent non-antagonistic interactions. (**b)** Heatmap representing the nested structure of the network. On the *X*-axis, sender strains (producers only) are sorted in the descending order of their respective sender degree. On the *Y*-axis, receiver strains (sensitive ones only) are sorted in the descending order of their respective receiver degree. Detailed representations of these heatmaps including the strain numbers and strain names are available in Supplementary Data S1a,b.

**Figure 2:**
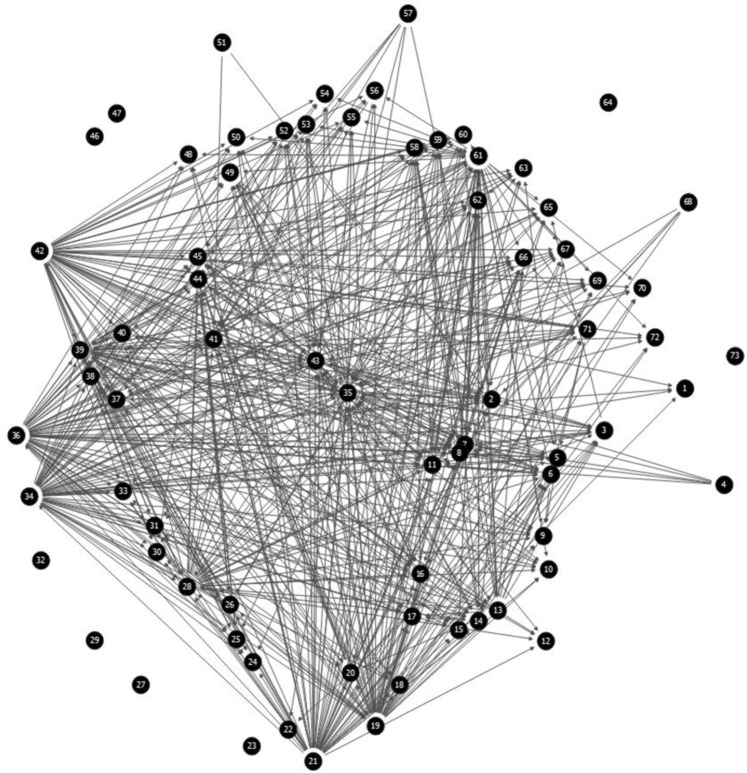
Network diagram of intraspecific competition among 73 strains of *C. maltaromaticum*. The “edges” depict directed inhibitory interactions and the “nodes” represent strains. The position of the “nodes”, from the border towards the center of the circular layout, is representative of an ascending receiver degree; the most sensitive strains being located near the center and the eight strains having null receiver degrees (interactive profile “R”) being located at the border.

In order to study competition among a collection of 73 strains of *C. maltaromaticum*, we set up an experimental system based on the use of high-throughput pairwise competition assays. For each individual pairwise interaction, one strain was put in the position of the sender strain and the other strain was put in the position of the receiver strain. The outcome of each individual pairwise interaction was determined by the impact of the Cell Free Supernatant (CFS) of one strain of the collection, called “sender strain”, on the growth of another strain of the collection, called “receiver strain”, which was cultivated in fresh medium (to avoid exploitation competition). The two possible outcomes of a pairwise interaction are the absence or the presence of inhibition, represented respectively by white and black squares in a heatmap compiling the results for the 5 329 pairwise interactions. Thus, the generated heatmap represents the competition network of *C. maltaromaticum* strains where the individual interaction potential of each strains can be deduced (Fig. 1a). The analysis of the network is based on the description of the ecological networks by Newman^21^; each black square of the heatmap represents an edge (inhibitory interaction) connecting two nodes (strains) in the network diagram (Fig. 2).

### General features of the competition network among *C. maltaromaticum* strains

Among 5 329 tested pairwise interactions, 574 antagonisctic interactions were observed (Fig. 1a). The interaction frequency was of 0.11. The resulting competition network connected all tested strains except eight strains that were not involved in any antagonistic interaction, neither as antagonizing strain, nor as antagonized strain. The network diameter was of 5, meaning that the most disconnected pair of strains were connected by 5 antagonistic interactions. Forty-one *C. maltaromaticum* sender strains were inhibiting at least one receiver strain, thus leading to a percentage of 56% producer strains (strains with status “P”). Furthermore, 60 *C. maltaromaticum* receiver strains were inhibited by at least one *C. maltaromaticum* sender strain (strains with status “S”), and therefore 82.2% of the strains are sensitive to at least one producer strain and 13 strains were resistant to all other strains. All these results show that intraspecific competition is high in *C. maltaromaticum*.

### Strain status according to production, sensitivity and resistance

**Figure 3:**
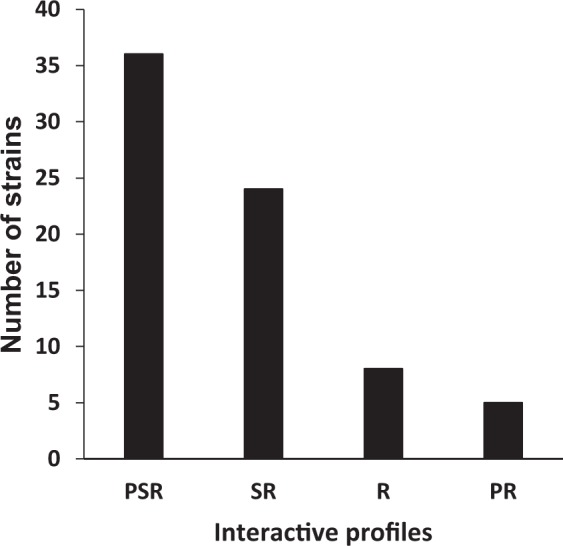
Interactive profiles of the 73 tested *C. maltaromaticum* strains. P means production of antimicrobial substances, S means sensitivity to antimicrobial substances produced by P strains, and R means resistance to antimicrobial substances produced by P strains.

According to the heatmap (Fig. 1a), one given strain can endorse several roles depending on the interacting strain and whether it is tested as a sender strain or as a receiver strain: therefore some strains may be able to produce one or several antimicrobial substances, and they may as well be sensitive but also resistant to various antimicrobial substances. These strains thus exhibit a PSR (Producer-Sensitive-Resistant) profile. The detailed analysis of the heatmap revealed that 36 strains exhibited a PSR profile, representing 49.3% of the collection (Fig. 3). These results thus show that approximately half of the strains are simultaneously producers of at least one growth-antagonizing substance able to inhibit at least one receiver strain, sensitive towards at least one producer strain, and resistant to at least one producer strain.

Besides PSR strains, 24 non-producer strains displayed sensitivity or resistance depending on the considered sender strain and were consequently assigned the SR (Sensitive-Resistant) profile. In addition, 8 non-producer strains that were resistant to all producers were assigned the R (Resistant) profile, and 5 strains which were producers and were resistant to all the other producers, were assigned the PR (Producer-Resistant) profile (Fig. 3).

### Sender and receiver degrees

Beyond the heterogeneity in the distribution of interactive profiles among strains, there was a large heterogeneity in the inhibitory activity spectra and sensitivity spectra of the strains, defined respectively as the sender degree and the receiver degree. Statistically, this heterogeneity was confirmed by calculating the sender-receiver asymmetry that is based on the variances of sender and receiver degrees. The sender-receiver asymmetry was equal to −0.38 indicating the network was sender-determined.

**Figure 4:**
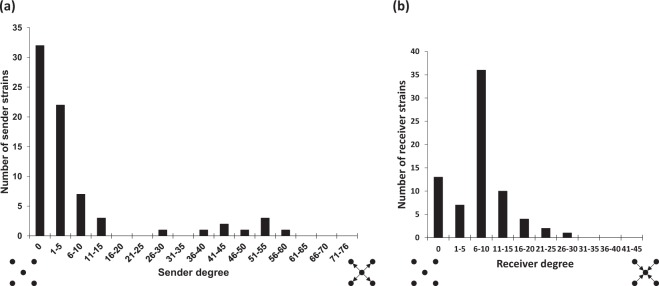
(**a**) Sender degree distribution: number of sender strains (*Y*-axis) as a function of the sender degree (*X*-axis), which is the number of receiver strains inhibited by a given sender strain. **(b)** Receiver degree distribution: number of receiver strains (*Y*-axis) as a function of the receiver degree (*X*-axis), which is the number of sender strains that inhibit one given receiver strain.

Sender strains displayed various sender degrees that vary from 1 to 56 inhibited strains (Fig. 4a). Under the conditions of evaluation, thirty-two sender strains exhibited a null sender degree and represented non-producer strains. Concerning the 41 producer strains, a majority of 32 strains (78% of the producers) inhibited between 1 and 25 strains, thus displaying relatively narrow inhibitory activity spectra. Relatively broad-spectra of inhibitory activity were displayed by 8 strains (19.5% of the producers), with a number of inhibited strains ranging from 36 to 56 sensitive strains per sender strain. It can be noticed that the 6*C. maltaromaticum* strains able to inhibit *L. monocytogenes* EGDe were among the strains having the highest sender degrees. Sensitive strains presented various sensitivity spectra (Fig. 4b). The distribution of the receiver degree was characterized by a bell shape. The overall population of sensitive strains displayed a narrow spectrum with a maximal peak (36 strains) for values of the receiver degree ranging from 6 to 10 (Fig. 4b). Overall, these results revealed that the majority of strains exhibited narrow spectra of inhibition and narrow spectra of sensitivity.

To test whether there is a relationship between the sender degree and the receiver degree, the heatmap was rearranged by removing the non producers in the sender axis, and by reordering the strains according to their sender and receiver degrees (Fig. 1b). The network was characterized by a strong nested pattern, statistically supported by the binmat method that attributed to the observed nestedness a score of 6.59 (p-value = 0). The nested pattern showed that sender strains with narrow spectrum tended to inhibit receiver strains exhibiting a broad sensitivity spectrum. Reciprocally, receiver strains with narrow sensitivity spectrum tended to be inhibited by strains having a broad inhibitory spectrum. Thus, antagonism was not randomly organized; the nested architecture of the heatmap implies that inhibition depended on two factors: the spectrum width of the inhibitory activity of the sender and the spectrum width of the receiver sensitivity.

### Reciprocity of inhibitory and non-inhibitory interactions

**Figure 5:**
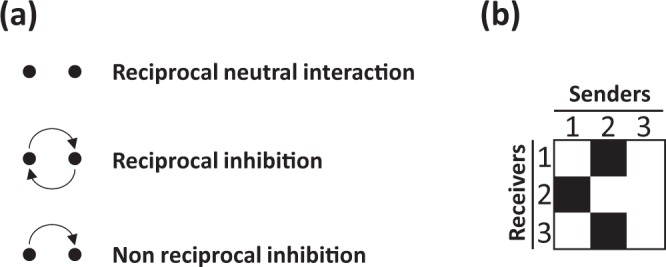
Definition of reciprocal and non reciprocal interactions. Reciprocal neutral interactions (**a**) refer to cases where no inhibition is observed between two strains. This is virtually exemplified by the pair of strains 1 and 3 (**b**): strain 1 does not inhibit strain 3, and reciprocally strain 3 does not inhibit strain 1. Reciprocal inhibition (**a**) refers to cases where mutual inhibitions are recorded as exemplified by the pair of strains 1 and 2 (**b**): strain 1 inhibits strain 2, and strain 2 inhibits strain 1. Non reciprocal inhibition (**a**) refers to cases where one strain inhibits the other strain without being inhibited by the latter. This is exemplified by the pair of strains 2 and 3 (**b**): strain 2 inhibits strain 3, and strain 3 does not inhibit strain 2.

Investigating the characteristics of the tested 5 329 pairwise interactions revealed the presence of three types of pairwise interactions: (i) reciprocal neutral interactions (no inhibition) between two strains, (ii) reciprocal inhibition between two strains and iii) non-reciprocal inhibition in which one strain inhibits the other strain without being inhibited by the latter (Fig. 5).

Reciprocal neutral interactions were the most frequent, characterizing 79.3% of total pairwise interactions. Non-reciprocal inhibitory interactions characterized approximatively 19,7% of pairwise interactions, whereas reciprocal inhibitory interactions were shown to be the less frequent, being represented by 48 out of 5 329 pairwise interactions, i.e., 0.9%.It can be noticed that reciprocal inhibitory interactions represent 8.4% of the 574 pairwise inhibitory interactions revealed by the competition assays. These reciprocal inhibitions consistently involve PSR strains. In total, 21*C. maltaromaticum* strains were part of reciprocal inhibitions and correspond simultaneously to a percentage of 51.2 of the producer strains (21 out of 41), and a percentage of 35 of the sensitive strains (21 out of 60).

Among the 21*C. maltaromaticum* strains involved in reciprocal inhibitions, only two strains were responsible for half the reciprocal inhibitions (24 out of a total of 48 reciprocal inhibitions).

## Methods

### Collection of bacterial strains

The *C. maltaromaticum* strain collection was built from several isolation campaigns which were performed between 1983 and 2010 (Supplementary Data S2). The 73 strains of the collection were isolated from diverse habitat. Among the 73 strains, 64 were isolated from different samples. Strains can be classified into 7 categories according to their respective habitats: 37 strains were isolated from dairy products, 22 were isolated from diseased fish, 2 were isolated from edible fish products, 6 were isolated from meat products, 3 were isolated from the environment, 2 were isolated from humans, and 1 is from an unknown origin.

Strains were cultivated in Trypticase Soy Broth medium (TSB) (bioMérieux, Marcy-l’Etoile, France) supplemented with 6 g/L of bacto-yeast extract (YE) and incubated at 30 °C for 16 h. Strains were stored in TSBYE medium supplemented with glycerol at a final concentration of 10% (v/v) at −80 °C and propagated twice before use. The receiver strains were cultivated in 96-well sterile microplates in which each well was filled with 195 µL TSBYE and inoculated with 5 µL of TSBYE cultures. The microplates were incubated for 24 h at 30 °C prior to storage at –80 °C. They were propagated once in fresh medium conditioned in 96-well microplates before use.

### Competition assays

For each high-throughput competition assay, a control microplate was designed in order to get measures of the growth kinetics of receiver strains in fresh TSBYE. Each competition assay was carried out following a three-days’ protocol. Two days prior to exposing the receiver strains to the CFS of sender strains, a colony of a sender strain was used to inoculate 10 mL TSBYE medium and the culture was performed by incubation at 30 °C for 24 h. The resulting culture was washed once with fresh TSBYE by centrifugation at 2 800 g for 10 min, then the cells were resuspended in 10 mL of fresh TSBYE medium. This suspension was diluted 1 000 times in 50 mL of TSBYE medium and was incubated at 30 °C for 24 h. In parallel, a microplate containing frozen cultures of receiver strains was thawed and 5 µL were used to inoculate 195 µL of fresh TSBYE in each well of a 96-well microplate which was subsequently incubated at 30 °C for 24 h. On day 3, CFS of each of the sender strain was prepared by centrifugation of the sender strain culture at 2 800 g for 10 min, followed by filtration of the supernatant through a 0.45 μm pore sized filter. In parallel, 20 µL of the receiver strains precultured in 96-well microplates were used to inoculate 90 µL of fresh TSBYE in 96-well microplate and then 100 µL of CFS of the sender strain was added to each well. A kinetic monitoring of the absorbance at 595 nm was carried out using a microplate reader (Infinite Pro 200, Tecan) integrated into an automated handling system allowing to incubate and monitor five 96-well microplates in parallel. For each batch of 5 microplates, one microplate was used as a growth positive control (in which 100 µL additional TSBYE medium was added instead of CFS), and the remaining microplates were used to test the effect of the CFS of 4 sender strains.

### High-throughput data processing

At the end of each run, data were exported to a spreadsheet. The data were processed by determining, for each kinetic, a quantitative Growth Inhibition Indicator (GII) that allowed to classify pairwise interactions into two categories: antagonistic and non-antagonistic interactions. The GII evaluates the impact of the sender strain CFS on the growth of the receiver strain by calculating the difference: t_2_- t_1_; where t_2_ is the time (min) required for the receiver strain culture to reach an OD_595nm_ value of 0.2 when cultivated under the presence of the CFS of the sender strain, and t_1_ is the time (min) required for the receiver strain to reach an OD_595nm_ of 0.2 when cultivated in fresh TSBYE medium exclusively. The threshold delineating the inhibition resulting from competition from non-inhibition outcomes, was determined by analyzing, for the whole collection, the impact of CFS of one given sender strain to itself as a receiver strain. All values were below 300 min which was therefore defined as the threshold (Supplementary Data S3). A small-scale duplication experiment was independently performed by reproducing 219 competition assays (involving 219 strain couples). Approximately 98.2% of the duplication results were reproducible (Supplementary Data S4).

The competition map was obtained by constructing an (73 × 73) adjacency matrix based on the GII values (Supplementary Data S5), then converting the latter into a binary matrix by assigning the number 1 to GII values higher than 300 and by assigning 0 to GII values lower than 300. The resulting binary matrix was converted into a heatmap. Receiver strains were interpreted as sensitive “S” when the GII value was higher than 300. Receiver strains exhibiting GII values lower than 300 were interpreted as resistant “R”. Sender strains whose CFS induced a GII value higher than 300 were interpreted as producers “P”. Hence, it was possible to obtain the individual interaction potential of each strain.

### Statistical network analysis

The network diagram compiling exclusively the directed inhibitory interactions as edges, and the bacterial strains as nodes, was obtained using the SocNetV software (Kalamaras D. *Social Network Visualizer* (SocNetV)) (Fig. 2). The interaction frequency of the resulting competition network was obtained by dividing the number of observed antagonistic interactions by the number of possible interactions^13^. This value ranges from 0, when none of the strains are competitive, to 1 when all strains compete with all strains. The network diameter, defined as the longest path connecting any two strains^21^, was obtained by the network analyzer tool implemented in Cytoscape software^22^.

Sender degrees and receiver degrees were calculated individually, for each strain, and correspond respectively to the number of strains inhibited by a given strain and the number of strains inhibiting it^15,21^. The sender-receiver asymmetry which identifies whether the competition network is determined on average more by sender strains or by receiver strains was calculated as proposed by Vetsigian *et al*. (2011): accordingly, the sender-receiver asymmetry is quantified by comparing the distribution of the sender degree with the distribution of isolates the receiver degree. Negative values are associated with sender-receiver asymmetry.

Nestedness was calculated as defined by Bascompte *et al*. (2003) using the algorithm of Rodriguez-Girones and Santamaria (2006); it designates a type of network architecture (topology) that is dictated by a specific arrangement of antagonistic interactions within the network formed by the total binary interactions. In the case of a competition network, nestedness implies that only sender strains displaying a wide spectrum of inhibitory activity - i.e., high sender degrees can inhibit receiver strains displaying a narrow spectrum of sensitivity – i.e., low receiver degrees. Reciprocally, only receiver strains displaying a wide spectrum of inhibition can be inhibited by sender strains exhibiting a narrow spectrum of inhibition.

One thousand random 73*73 adjacency matrices were simulated as follows. Each matrix was simulated with the same proportion of inhibitory interactions as our observed matrix (574 inhibitory interactions out of a total of 5 329). In the adjacency matrices, the inhibitory interactions were represented by the number “1”, whereas the non-inhibitory interactions were represented by the number 0. For each of these 1 000 simulated matrices, 574 “1” were randomly placed in the matrix, outside the diagonal, the other inputs of the matrix were “0”. We then calculated the nestedness in all random matrices. The 1000 scores of nestedness obtained were between 29.95 and 37.5. Since the p-value is defined by the proportion of simulated matrices with a nestedness lower than the nestedness measured on our observed matrix, we deduced that the p-value is zero.

## Discussion

In this study, intraspecific competition in *C. maltaromaticum* was mapped by performing high-throughput competition assays which investigated a total of 5 329 pairwise interactions within a collection of 73 strains. A high level of competition, at the intraspecific level, was recorded with approximatively 56% of the sender strains inhibiting at least one receiver strain and 82.2% of the receiver strains being inhibited by at least one sender strain. A previous study mapped interference competition at the interspecific level among 185 *Vibrionaceae* strains and revealed that 44% of the sender strains inhibited at least one other strain and 86% of the receiver strains were inhibited by at least one strain^23^. A more recent study mapped intergeneric interference competition within a collection of 37 gamma-proteobacteria belonging to different genera and showed that 81% of the sender strains antagonized at least one other strain and 81% of the receiver strains were antagonized by at least one strain^12^. This suggests that competition at the intraspecific level in *C. maltaromaticum* is relatively high because percentages of both antagonizing strains were higher within *C. maltaromaticum* population compared to the recorded percentages in the population of *Vibrionaceae*. Furthermore, when compared to intergeneric interference competition among gamma-proteobacteria, intraspecific competition in *C. maltaromaticum* was characterized by a slightly higher percentage of antagonized strains (82.2% of antagonized strains for *C. maltaromaticum*, and 81% of antagonized strains for gamma-proteobacteria). A high proportion of antagonism in *C. maltaromaticum* could be due to the fact that *C. maltaromaticum* strains were not collected from a same community. The majority of the strains were indeed isolated from different products (64 out of 73, see Supplementary Data S2). In *Bacillus* spp., the frequency of antagonism is indeed higher between bacteria isolated from different sites than bacteria isolated from the same site and was interpreted as a higher level of sympatric resistance^15^.

Besides the impact of geographic partitionning of the strains, the intraspecific phylogenetic scale and niche overlap could also explain the high frequency of antagonisms in *C. maltaromaticum*. Russel *et al*., 2017, by mapping interference competition within a collection of 67 bacterial species belonging to different phyla, demonstrated that inhibition was more frequent between phylogenetically related species characterized by a niche overlap. According to the authors, the main driver of competition seemed to be metabolic similarity that is the degree of niche overlap between the interacting microorganisms. The high level of competition obtained in *C. maltaromaticum* is in accordance with this theory since strains of this species have highly similar metabolism^24^. In line with this, numerous strains of *C. maltaromaticum* produce bacteriocins with anti-*Listeria monocytogenes* activity. This high frequency of anti-*Listeria* strains in this species is consistent with the fact that these species occupy similar niches: both species colonize food products such as dairy, meat and fish product, and both are psychrophilic bacteria.

*C. maltaromaticum* strains could be categorized into four different interactive profiles: PSR, SR, PR and R. About half of the strains (49.3%) exhibited a PSR profile while the PR profile was the rarest. Interestingly, modelling studies suggest that one important condition to meet in order to sustain microbial diversity despite interference competition is that all actors should exhibit a PSR profile^10^. However, this was not observed for all species. Prasad *et al*. 2011 were interested in delineating interacting strains of arctic bacteria according to their interactive profiles. In their study, they mapped antagonism among 139 strains isolated from five soil samples and they categorized strains of the four samples in which antagonism was observed. Seven different interactive profiles were identified, thus leading to a higher level of complexity compared to our study that lacks three profiles that were additionally observed within the collection of arctic bacteria: PS, P and S. Moreover, their results highly contrasted with the results obtained in this study, since the PSR profile was the less represented in all of the studied samples (the rarest in three samples and absent in one sample), whereas the R profile was the most common in two samples and the second most common in the other two samples. The reason explaining these differences is still unknown, future studies will be required to understand how microbial populations are shaped regarding these interacting profiles.

The competition network of *C. maltaromaticum* revealed a high variability of both the sender degrees characterizing the antagonistic activity spectra of sender strains and the receiver degrees characterizing the sensitivity spectra of receiver strains. Indeed, the sender degree is broad, ranging from 0 to 56 antagonized strains and peaks near extreme low values with 78% of the sender strains displaying low sender degrees (between 1 and 15). By contrast, the receiver degree is narrower than the sender degree, ranging from 0 to 27 antagonizing strains, and has an unimodal pattern. It was previously demonstrated for the competition network among 64 *Streptomyces* strains that the sender degree peaks near both extreme values, meaning that the majority of the sender strains are either slightly antagonistic or highly antagonistic^13^. This contrasts with the low frequency of highly antagonistic strains in *C. maltaromaticum*. This difference between *Streptomyces* and *C. maltaromaticum* could be explained by their different antagonistic potentialities; the genes encoding the antagonistic substances in *Streptomyces* genomes are more numerous and of a different type than those encoded by the species *C. maltaromaticum* and by lactic bacteria in general. *Streptomyces* produce various types of antimicrobial secondary metabolites including non ribosomal peptides, polyketones and bacteriocins^25^ while for *C. maltaromaticum* species, studies mainly characterized bacteriocins^26,27^. As for the distribution of receiver degrees, a similar narrow and unimodal pattern was observed in *Streptomyces* competition network, meaning that in *C. maltaromaticum* and *Streptomyces*, sensitive strains tended to have intermediate sensitivity levels, few were highly antagonized. Nevertheless, the sender-receiver asymmetry coefficient was very similar in both competition networks with a value of −0.38 in *C. maltaromaticum* and a value of −0.37 in *Streptomyces*. This implies that the outcome of competition is determined by the sender strains rather than the receiver strains. Surprisingly, the mapped competition among the 37 gamma-proteobacteria was also characterized by a sender-receiver asymmetry estimated by a value of – 0.31, very close to the previous ones^12^. This suggests that the sender determined asymmetry could be a common property of bacterial competition at many taxonomic levels, since it has been demonstrated for intergeneric competition^12^, interspecific competition^13^ and, by this present study, for intraspecific competition. More data should be generated from more bacterial competition network in order to test this hypothesis.

Sender-receiver asymmetry was found in *C. maltaromaticum*. In ecological terms, Vetsigian *et al*. (2011) stated that a sender-receiver asymmetry implies that the existence of an inhibitory interaction is more influenced by the sender which needs to produce an antagonistic substance than by the receiver which doesn’t need to resist to it. However, the analysis of *C. maltaromaticum* competition network revealed that antagonistic interactions are shaped by a strong pattern of nestedness (with a score of 6.59). This nestedness pattern indicates that a double determinism dictates the outcome of competition which depends on the the width of inhibition spectrum of sender strains and the width of sensitivity spectrum of the receiver strains. Similarly, Aguirre-von-Wobeser *et al*. 2014 were able to elucidate a nested structure of interference competition among 37 gamma-proteobacteria^12^ with a score of 0.872. The double determinism of inhibition implies that the competition is not randomly structured and the outcome of competition is related to the typology of both the sender and the receiver in terms of spectrum width.

The study of the reciprocity of the interactions in *C. maltaromaticum* revealed that reciprocal neutrality was the most frequent case (79.3% of total pairwise interactions). Approximately 20% percent of the remaining pairwise interactions were non-reciprocal inhibitory interactions, whereas only a minority were reciprocal inhibitory interactions (0.9% of total pairwise interactions). Such knowledge could be very useful for the selection of strains of this species in food production. Indeed, industrial food fermentation involves the use of industrial cultures. In the dairy industry for instance, milk is inoculated with cultures of selected microorganisms which act as major contributors of milk transformation to cheese or other fermented dairy products. These cultures often contain several different microorganisms. One major issue in this field of industrial culture is the assembly of these different microorganisms in order to get a synthetic community where each microorganism can cohabit. This requires reciprocal neutral interactions between the selected strains. Our results allowed us to identify strains exhibiting low potential of mutual inhibition in our experimental conditions. These strains are predicted to exhibit a high compatibility degree and could be co-cultivated without strong mutual competitive exclusion. These strains would be good candidates for the design of mixed starters.

This study revealed the extent and the structure of competition network in a highly genetically diversified LAB species. However, the study was conducted under laboratory conditions that poorly mime the natural conditions. It is therefore not known to what extent these competition interactions occur in ecosystems and to what extent the individual interaction profiles of strains could vary in a natural setting. Future studies are required to unravel the extent of interference competition in *C. maltaromaticum* in experimental models which take into account factors such as the the expression regulation of genes encoding antimicrobial substances, the stability and the diffusion of the secreted antimicrobial substances in the matrices.

Our study assessed competition that likely involves diffusible antimicrobial substances since the receiver strains were exposed to cell-free supernatant of sender strains. However, interference competition can also involve cell-cell contact^28^. It would also be interesting to study the extent of competition phenomena involving cell-cell contact and the structure of the resulting competition networks.

In addition, further studies of interspecific competition between diverse microbial species are needed in order to elucidate the role of the competition in microbial community assembly.

## Supplementary information

Dataset 1.

Dataset 2.

Dataset 3.

Dataset 4.

Dataset 5.
